# Attenuation of VEGFR-2 Expression by sFlt-1 and Low Oxygen in Human Placenta

**DOI:** 10.1371/journal.pone.0081176

**Published:** 2013-11-19

**Authors:** Ori Nevo, Dennis K. Lee, Isabella Caniggia

**Affiliations:** 1 Department of Obstetrics and Gynecology, Sunnybrook Health Sciences Centre, University of Toronto, Toronto, Ontario, Canada; 2 Department of Obstetrics and Gynecology, Mount Sinai Hospital, Samuel Lunenfeld Research Institute, Toronto, Ontario, Canada; 3 Department of Physiology, University of Toronto, Toronto, Ontario, Canada; VU University Medical Center, Netherlands

## Abstract

Vascular endothelial growth factor receptor 2 (VEGFR-2), the primary receptor for VEGF, is crucial for normal endothelial function. sFlt-1, a truncated and soluble form of VEGFR-1 which binds and inhibits VEGF, is increased in preeclampsia and is positively regulated by low oxygen. Here, we investigated the effects of sFlt-1 and hypoxia on VEGFR-2 expression and signaling in the human placenta. VEGFR-2 transcript and protein levels were significantly decreased in preeclamptic placentae compared to controls (1.82 and 1.85 fold, respectively). An inverse correlation was observed for VEGFR-2 and sFlt-1 levels in both singleton and twin placentae from patients with preeclampsia. Immunofluorescence analyses revealed co-localization of VEGFR-2 and sFlt-1 in placental vasculature and co-immunoprecipitation analyses confirmed VEGFR-2 and sFlt-1 interaction only in preeclamptic placentae compared to age-matched controls. VEGFR-2 transcript and protein levels from explants cultured in 3% O_2_ were significantly decreased compared to those incubated at 20% O_2_ (5.9 and 12.47 fold, respectively). Also, VEGFR-2 transcript levels were significantly decreased in early first trimester placentae (low oxygen environment) compared to late first trimester placentae (2.05 fold). We next explored whether sFlt-1 directly affects VEGFR-2 expression. Treatment of first trimester placental explants with sFlt-1 resulted in significantly decreased levels of VEGFR-2 (2.03 fold) and downstream signaling proteins phospho-ERK (1.60 fold) and phospho-Akt (1.64 fold). Our findings show a novel hypoxia-induced and PE-related down-regulation of VEGFR-2 in the human placenta. sFlt-1, which is known to be increased in hypoxic conditions and PE, directly attenuates VEGFR-2 expression and signaling. A direct interaction between sFlt-1 and VEGFR-2 may represent an important mechanism in VEGFR-2 regulation, inhibition of VEGFR-2-mediated processes in placentation and a novel platform to examine the onset of preeclampsia.

## Introduction

Preeclampsia is a severe complication of pregnancy characterised by increased maternal blood pressure, proteinuria, and end organ disease due to systemic vascular/endothelial dysfunction. In recent years, soluble VEGF receptor-1 (sFlt-1) has been recognized as an important factor that is involved in the pathogenesis of preeclampsia through inducing vascular dysfunction [1]. It has been shown that placentae from patients with severe preeclampsia secrete excess sFlt-1 accompanied by markedly increased maternal sFlt-1 serums levels [1]. The primary mechanism by which sFlt-1 alters angiogenesis and induces endothelial dysfunction is thought to be by sequestering its endogenous ligands thus decreasing their bioavailability. sFlt-1 is a truncated splice variant of VEGF receptor-1 (VEGFR-1), a transmembrane receptor tyrosine kinase that binds both vascular endothelial growth factor (VEGF) and placental growth factor (PlGF) with high affinity. The function of the full-length VEGFR-1 is not completely clear, however, it has been shown to be important for normal vascular development and branching angiogenesis [2]. sFlt-1 consists of six extracellular IgG like domains with a unique C-terminus, lacking the transmembrane and intracellular domains. It has been reported that in-vivo, sFlt-1 exists as several isoforms with various molecular weights between 100 and 145 kDa [3], [4].

The primary receptor for VEGF is VEGF receptor-2 (VEGFR-2, also known as KDR) which is essential for normal endothelial proliferation and vascular formation. VEGFR-2 is a transmembrane receptor that is comprised of 7 extracellular IgG like domains, a membrane-spanning domain and an intracellular kinase domain [5]. Activation of VEGFR-2 occurs when the VEGF dimer binds to VEGFR-2 domains 2 and 3 and facilitates receptor dimerization which in turns activates its intracellular kinase domains. VEGFR-2 typically forms homodimers, though heterodimers with VEGFR-1 have been reported [6]–[8]. VEGFR-2 is expressed in endothelial cells, megakaryocytes and haematopoietic stem cells [9] and also in the trophoblast layer in human placenta [10]. Zhou et al. reported that VEGFR-2 is localized to cytotrophoblasts during the first trimester and to the proximal column of proliferating cytotrophoblasts with only low expression in extravillous invading trophoblasts [10]. Though the specific function of VEGFR-2 in trophoblasts is not clear it may be important for their transformation into intravascular trophoblasts and transforming the spiral arteries [10].

VEGFR-2 expression in endothelial cells has been reported to be regulated by various conditions and, interestingly, low oxygen, which promotes angiogenesis and has been linked to reduced VEGFR-2 expression through a yet undefined mechanism [11], [12]. However, the regulation of VEGFR-2 expression in the human placenta that also expresses high levels of sFlt-1 has not been reported yet.

Our aim here was to explore the role of sFlt-1 and hypoxia in VEGFR-2 expression in the human placenta during normal and preeclamptic pregnancies.

## Materials and Methods

### Tissue collection

The study was approved by the Research Ethics Board of Sunnybrook Health Sciences Centre and written informed consent from all participants was obtained. First trimester placental samples were collected from elective first-trimester pregnancy terminations performed by dilatation and curettage. All women were healthy non-smokers, with no previous history of preeclampsia, diabetes, essential hypertension or renal disease. Preterm and term placental samples (control, preeclamptic, control twins and preeclamptic dichorionic twins) were collected immediately after delivery and snap frozen as previously described [13]. The preeclamptic group was selected to represent classic severe early-onset (less than 34 weeks) preeclampsia according to both clinical and laboratory findings based on the American College of Obstetrics and Gynecology criteria [14]. Women in the control groups did not show clinical or pathological signs of preeclampsia, infections or other maternal or placental disease. Gestational age was determined by the date of the last menstrual period and first trimester ultrasound measurement of crown rump length.

### First-trimester human chorionic villous explants culture

Villous explants cultures were established from first trimester human placentas (6 to 12 weeks of gestation) obtained from elective terminations of pregnancies as previously described [13]. Explants were maintained in either a standard condition of 20% O_2_ (5% CO_2_ in 95% air) or under relative hypoxic conditions of 3% O_2_ (5% CO_2_ in 92% N_2_) for up to 24 hours at 37°C (n = 7). It is noteworthy that 3% O_2_ levels are physiologically appropriate for early first trimester placentae, and is considered hypoxic for the purposes of this experiment relative to the 20% O_2_ standard. In a separate experiment, the effects of sFlt-1 on explants cultures obtained from placentas 6 to 9 weeks of gestational age were assessed. Explants were incubated in standard conditions in the presence or absence of 500 ng/mL of sFlt-1 (soluble VEGFR1, Cell Sciences, Canton, MA) or 600 ng/mL of a neutralizing antibody to sFlt-1 (AF321, R&D Systems, Minneapolis, MN) overnight (17 hours) at 37°C (n = 3).

### RNA isolation and quantitation using real-time PCR (qPCR)

RNA isolation and qPCR were performed as previously described [13]. Briefly, total RNA was extracted from placental tissues using TRIzol reagent (Invitrogen, Carlsbad, CA), treated with DNase (Ambion, Austin, TX) to remove genomic DNA contamination and further purified by column purification (Qiagen, Valencia, CA). 1 µg of total RNA was reverse transcribed using random hexamers (Applied Biosystems, Inc., Foster City, CA). The resulting templates were quantified by real-time PCR (MJ Research Inc., Waltham, MA) as previously described [15]. TaqMan primers and probe for VEGFR-2 and 18S were purchased from Integrated DNA Technologies (PrimeTime Mini qPCR Assay N002253.1.pt.KDR) and Applied Biosystems, Inc. respectively. Relative quantification of data was performed using logarithmic curves. Expression levels of VEGFR-2 were normalized with 18S expression using the 2ΔΔCt formula as previously described [15].

### Western blot analysis

Western analyses were performed as previously described [13]. Briefly, 50 µg of total protein lysates were subjected to 8% (w/v) sodium dodecyl sulphate-polyacrylamide gel electrophoresis. Following electrophoresis, proteins were transferred to polyvinylidene difluoride membranes. Non-specific binding was blocked by incubation in 5% (w/v) nonfat dry milk in Tris-buffered saline containing 0.1% (v/v) Tween-20 (TBST) for 60 minutes. Membranes were incubated with antibodies specific for VEGFR-2 (#2479, Cell Signaling Technology, Beverly, MA), sFlt-1 (AF321, R&D Systems), phospho-Erk1/2 (#9106, Cell Signaling Technology), phospho-Akt (#4060, Cell Signaling Technology) or actin (sc-1616, Santa Cruz Biotechnology, Santa Cruz, CA) in 5% milk or 5% bovine serum albumin (as per manufacturer's instructions) at 4°C overnight. Membranes were washed in TBST and incubated for 60 minutes at room temperature with 1:2000 diluted horseradish peroxidise-conjugated anti-rabbit, goat or mouse IgG (Santa Cruz Biotechnology) in 5% milk in TBST. After washing with TBST, blots were visualized by enhanced chemiluminescence substrate Western Lightning Plus-ECL (Perkin Elmer, Waltham, MA). Protein levels were determined by densitometric analyses of specific bands using ImageJ software (U.S. National Institutes of Health, Bethesda, Maryland) and normalized against actin or Ponceau-stained standards.

### Immunofluorescence

Immunofluorescence analyses were performed as previously described [16]. Briefly, fixed tissue sections embedded in paraffin were mounted and prepared on glass slides. After deparafinization, sodium citrate retrieval was performed followed by treatment with sudan black (0.1% sudan black in 70% ethanol) to quench endogenous fluorescence. Sections were blocked with 5% normal horse serum for 1 h, and incubated overnight with primary antibodies for sFlt-1, 1:150 dilution (Zymed Laboratories, San Fransisco, CA) and VEGFR-2, 1:200 dilution (Santa Cruz biotechnology). After washings, the slides were incubated with secondary antibodies at 1:300 dilution (Alexa fluor) and then treated with 0.4% DAPI for nuclear detection. Negative control conditions were performed by omitting primary antibodies and incubating the slides with rabbit or mouse IgG.

### Immunoprecipitation analysis

Immunoprecipitation protocols were performed as described previously [17]. Briefly, 500 µg protein samples were precleared with 20 µL of Protein-A Agarose (sc-2001, Santa Cruz Biotechnology), centrifuged and supernatants incubated with 1 µg of VEGFR-2 antibody (Cell Signaling Technology) rotating overnight at 4°C. 20 µL of Protein-A Agarose was added to the mixture and further incubated 30 minutes at 4°C. Precipitates were collected by centrifugation, washed 3 times in ice cold RIPA buffer and resuspended in 1X SDS loading buffer. The samples were incubated at 95°C for 5 minutes and analysed by western analysis.

### Statistics

Statistical analyses were performed using GraphPad Prism software (San Diego, CA). For comparison of data between multiple groups, we used one-way ANOVA with post hoc Dunnet test. For comparison between two groups, we used the Mann-Whitney test and paired or unpaired t-test when applicable. Significance was defined as *P*<0.05. Results are expressed as the mean±SEM (SE).

## Results

### VEGFR-2 expression levels are decreased in preeclamptic placentae and are inversely correlated with sFlt-1

**Figure 1:**
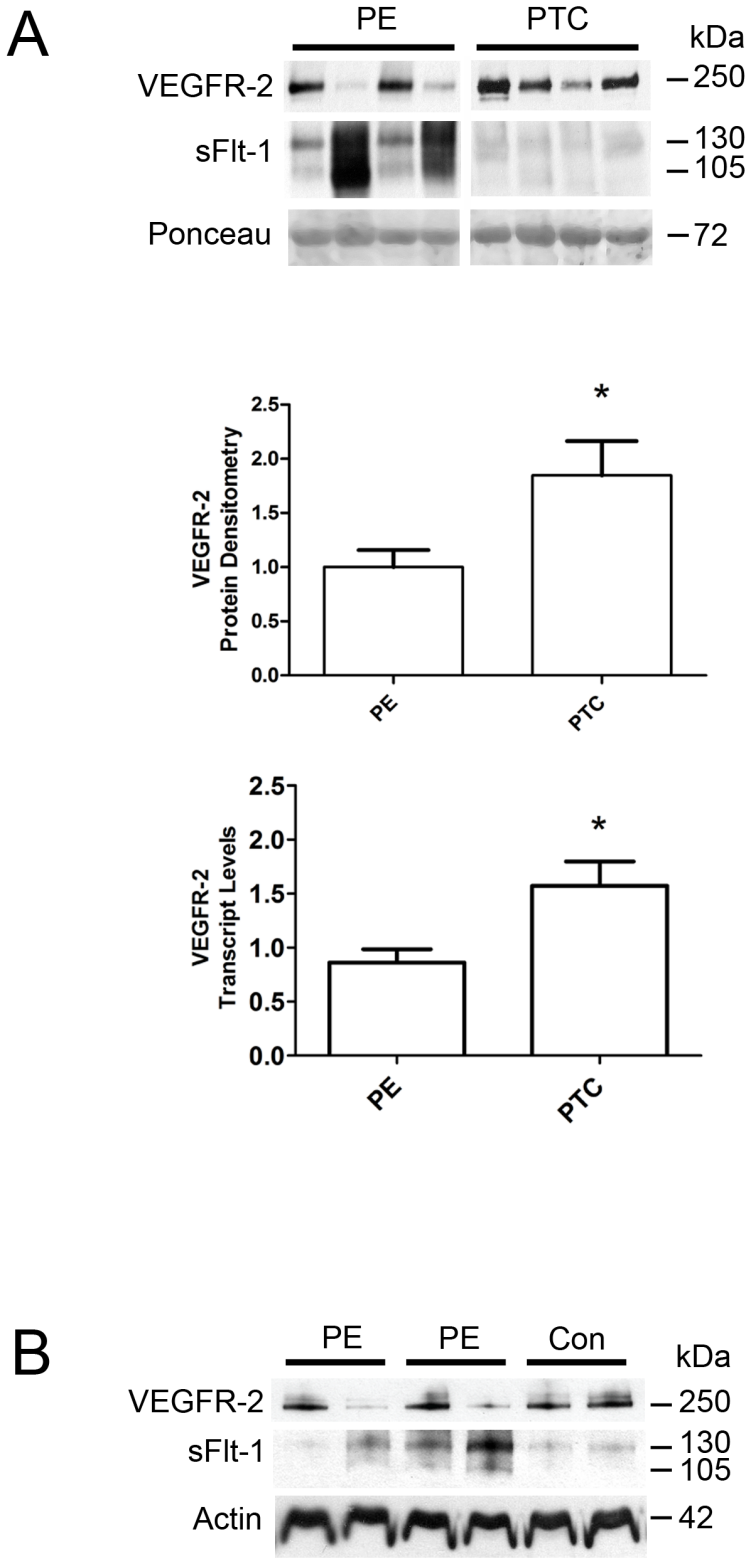
An inverse correlation between VEGFR-2 and sFlt-1 levels in preeclamptic placentae. **A:** Representative VEGFR-2 and sFlt-1 immunoblots of PE and PTC placentae. Densitometry analysis comparing normalized VEGFR-2 immunoreactivity between PE (n = 13) and PTC (n = 8) samples. VEGFR-2 transcript levels were assessed by qPCR analysis comparing PE (n = 12) and PTC (n = 7) samples. **B:** Representative VEGFR-2 and sFlt-1 immunoblots of placental tissues from PE and control (Con) dichorionic twins. (PE, preeclampsia; PTC, age-matched preterm control; *P<0.05). All values are represented as the means±SEM.

Recent reports have shown that preeclampsia (PE) is associated with elevated levels of sFlt-1, in both the placenta and in the maternal circulation, with subsequent repercussions in maternal vascular dysfunction. As the primary receptor for VEGF and target for VEGF-mediated regulation of vascular homeostasis, we hypothesized that VEGFR-2 would also exhibit altered expression levels in PE placentae. We first compared the expression levels of VEGFR-2 in both PE and age-matched preterm control (PTC) placentae (Fig. 1A). Protein lysates were prepared from frozen placental tissue samples and subjected to western analysis. VEGFR-2 protein levels were significantly decreased 1.85 fold in PE compared to PTC placentae (1.00±0.16 versus 1.85±0.32, *P*<0.05). In addition, VEGFR-2 transcript levels were also measured by qPCR analysis in PE and PTC placental tissues. VEGFR-2 transcript levels were significantly decreased 1.82 fold in PE compared with PTC placentae (0.86±0.12 versus 1.57±0.23, *P*<0.05), in close approximation with the differences observed at the protein level. Together, these findings confirmed a decreased level of VEGFR-2 expression in PE placentae.

Coupled with previous findings of increased expression of sFlt-1 [13], the decreased expression of VEGFR-2 in PE placentae suggests VEGFR-2 and sFlt-1 share an inverse correlation in expression levels. Our western analyses confirm this hypothesis, as PE placenta samples favor the expression of either VEGFR-2 or sFlt-1 over the other (Fig. 1A). In addition, we also performed western analyses on placental tissues from dichorionic preeclamptic twin pregnancies and from dichorionic control twin pregnancies, as previously described [18]. Compared to control placentae, which exhibited similar expression levels of VEGFR-2 and sFlt-1 in the two placentae, the preeclamptic placentae revealed extensive differences in expression levels, with one placenta favoring high levels of VEGFR-2 and lower level of sFlt-1 compared to the other twin placenta which favored increased levels of sFlt-1 and lower level of VEGFR-2 (Fig. 1B).

### VEGFR-2 expression is decreased in hypoxic placental explants and first trimester placentae

**Figure 2:**
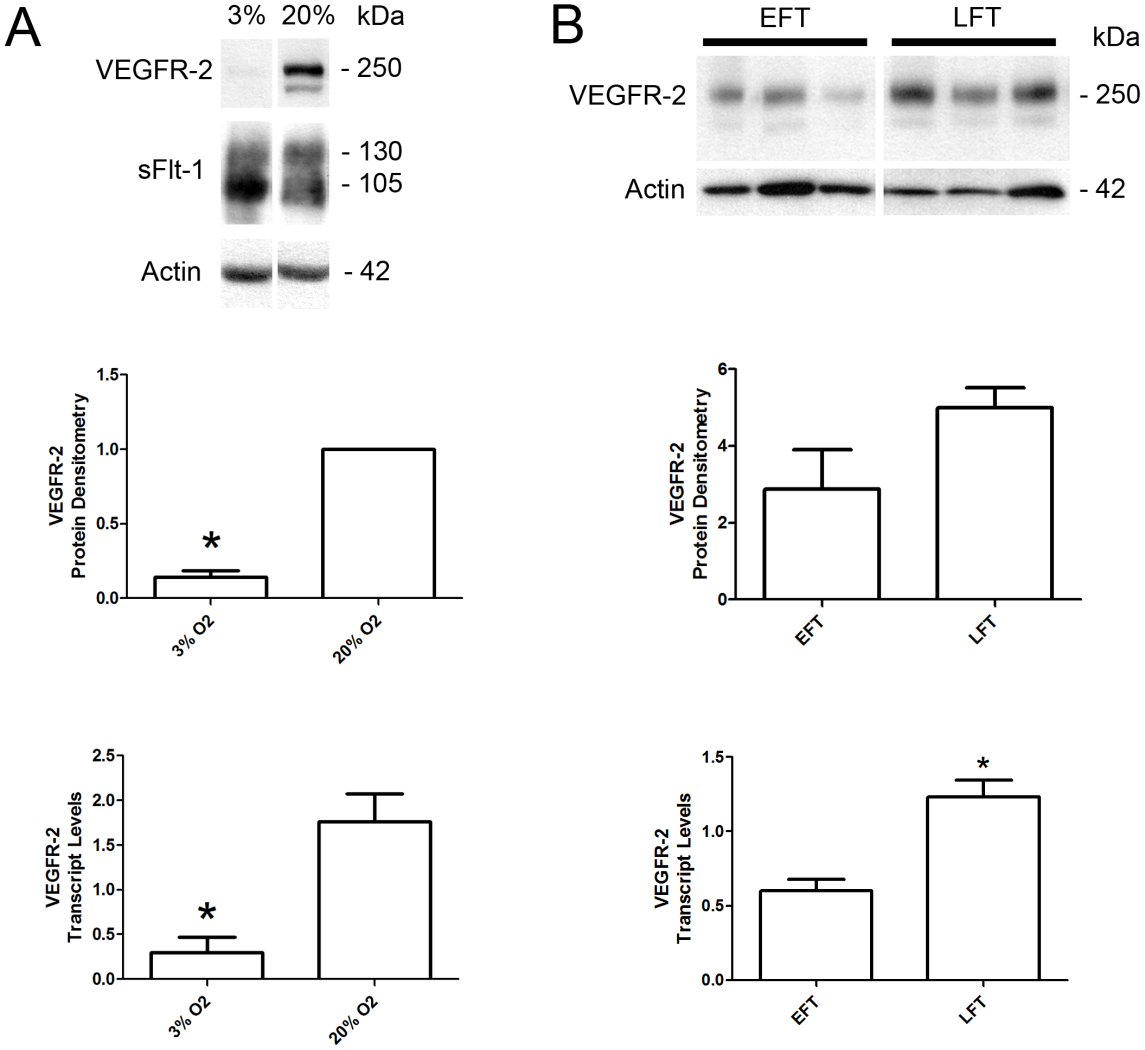
Decreased levels of VEGFR-2 in hypoxia and in early first trimester placentae. **A:** Representative VEGFR-2 and sFlt-1 immunoblots of placental villous explants cultured at 3% and 20% O_2_. Densitometry analysis comparing normalized VEGFR-2 immunoreactivity between explants cultured at 3% (n = 7) and 20% O_2_ (n = 7). VEGFR-2 transcript levels were assessed by qPCR analysis comparing 3% (n = 4) and 20% O_2_ (n = 4) samples. **B:** Representative VEGFR-2 immunoblots of early (EFT) and late (LFT) first trimester placentae. Densitometry analysis comparing normalized VEGFR-2 immunoreactivity between samples were not significantly different. Transcript levels were assessed by qPCR analysis comparing VEGFR-2 levels in EFT and LFT placentae. (EFT, n = 12; LFT, n = 14; *P<0.05). All values are represented as the means±SEM.

Our previous work examined the effect of hypoxia on sFlt-1 levels in placentae, and observed a HIF-1-mediated increase in sFlt-1 with decreased oxygen levels[13]. Given the inverse levels of VEGFR-2 and sFlt-1 observed in PE samples, we investigated whether oxygen levels also played a role in VEGFR-2 expression levels. Protein lysates and purified RNA were prepared from first trimester placental villous explants cultured in 3% and 20% O_2_ and subjected to western and qPCR analyses respectively (Fig. 2A). VEGFR-2 protein levels were significantly decreased 12.47±2.84 fold (*P*<0.05) in explants cultured in 3% O_2_ compared to 20% O_2_. VEGFR-2 transcript levels were similarly decreased 5.9 fold in 3% O_2_ explants compared to 20% O_2_ explants (0.30±0.17 versus 1.76±0.31, *P*<0.05). In addition, western analysis of these samples confirmed our previous findings of increased sFlt-1 levels in explants cultured under hypoxic conditions [13]. Together, these findings show that unlike sFlt-1 expression, VEGFR-2 expression is markedly decreased under low oxygen conditions in the human placenta.

During the transition from early first trimester of pregnancy (first 10 weeks of gestation) to the late first trimester (11 to 14 weeks of gestation), the oxygenation of the placenta undergoes a surge as the intervillous space opens to the maternal circulation [19]. Therefore, we hypothesized a marked increase in VEGFR-2 expression over this period of development. Western blot analysis of placental lysates from early and late first trimester revealed lower levels of VEGFR-2 during the early first trimester (Fig. 2B). Purified RNA was prepared from frozen placental tissues collected from early and late first trimester. qPCR analysis revealed that the VEGFR-2 transcript levels were significantly decreased 2.05 fold in placentae from early first trimester samples compared to later first trimester samples (0.60±0.08 versus 1.23±0.11, *P*<0.05), confirming a marked increase in VEGFR-2 expression coinciding with increased oxygenation in-vivo (Fig. 2B).

### Spatial localization of VEGFR-2 in preeclamptic and control placentae

**Figure 3:**
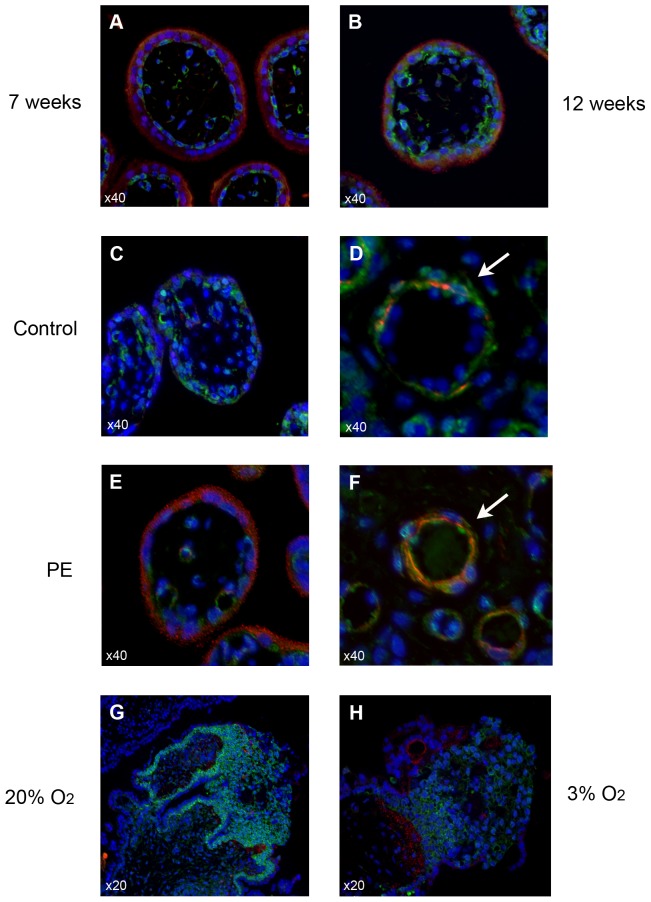
Immunofluorescence localization of sFlt-1 (red) and VEGFR-2 (green). Nuclear localization by DAPI staining is shown in blue. **A:** 7 weeks placenta **B:** 12 week placenta. **C–D:** control. **E–F:** early preeclampsia. **G:** first trimester villous explants at 20% oxygen and **H:** villous explants at 3% oxygen. Co-immunofluorescence is shown in yellow/orange. White arrow: vessel wall.

The localization and function of the VEGFR-2 receptor have been best characterized in the endothelial layer of blood vessels. However, recent studies have also determined localization of VEGFR-2 in placental trophoblasts [10]. To elucidate variations in the spatial localizations of VEGFR-2 and sFlt-1 in human placentae, immunofluorescence (IF) analyses of paraffin-embedded sections of fixed placental tissue were performed (Fig. 3). We first examined temporal differences of VEGFR-2 and sFlt-1 spatial localizations in first trimester placentae. In a series of placental tissue sections ranging from early to late first trimester, strong immunoreactivity for sFlt-1 was observed in the syncytiotrophoblast layer of placental villi in early first trimester samples, while the underlying cytotrophoblast layer and vascular regions exhibited weak immunoreactivity for VEGFR-2 (Fig. 3A). In contrast, cross-sections of late first trimester placental villi revealed a marked decrease in sFlt-1 levels in the syncytiotrophoblast layer and overall increase in VEGFR-2 expression in cytotrophoblasts and vessels (Fig. 3B).

We next examined the spatial localizations of VEGFR-2 and sFlt-1 in control and PE placentae. The distributions of VEGFR-2 and sFlt-1 in control placental villi more closely resembled the highly oxygenated late first trimester placentae, with greatly decreased levels of sFlt-1 in the syncytiotrophoblast layer (Fig. 3C) and weak co-localization of VEGFR-2 and sFlt-1 in vessels (Fig. 3D). By contrast, the distribution of VEGFR-2 and sFlt-1 in PE placental villi was remarkably similar to the patterns observed in younger, less oxygenated early first trimester placentae (Fig. 3E). sFlt-1 expression was strongest in the syncytiotrophoblast layer, overlaying a weak positive immunoreactivity for VEGFR-2 in trophoblasts and in vessels. Strong co-immunofluorescence for sFlt-1 and VEGFR-2 was noted in vessels from PE placentae (Fig. 3F). We also examined the localization of VEGFR-2 expression in villous explants. Sections of explants exposed to 20% oxygen (Fig. 3G) showed increased VEGFR-2 immunofluorescence in the cytotrophoblast layer and in the proximal column of proliferating extravillous trophoblasts compared to 3% oxygen (Fig. 3H). Overall, our immunofluorescence data shows that VEGFR-2 is expressed in cytotrophoblasts, proliferating trophoblasts and placental villous vasculature. In preeclampsia, VEGFR-2 expression is reduced while vascular co-localization with sFlt-1 is prominent.

### sFlt-1 interaction with VEGFR-2 is increased in preeclampsia

**Figure 4:**
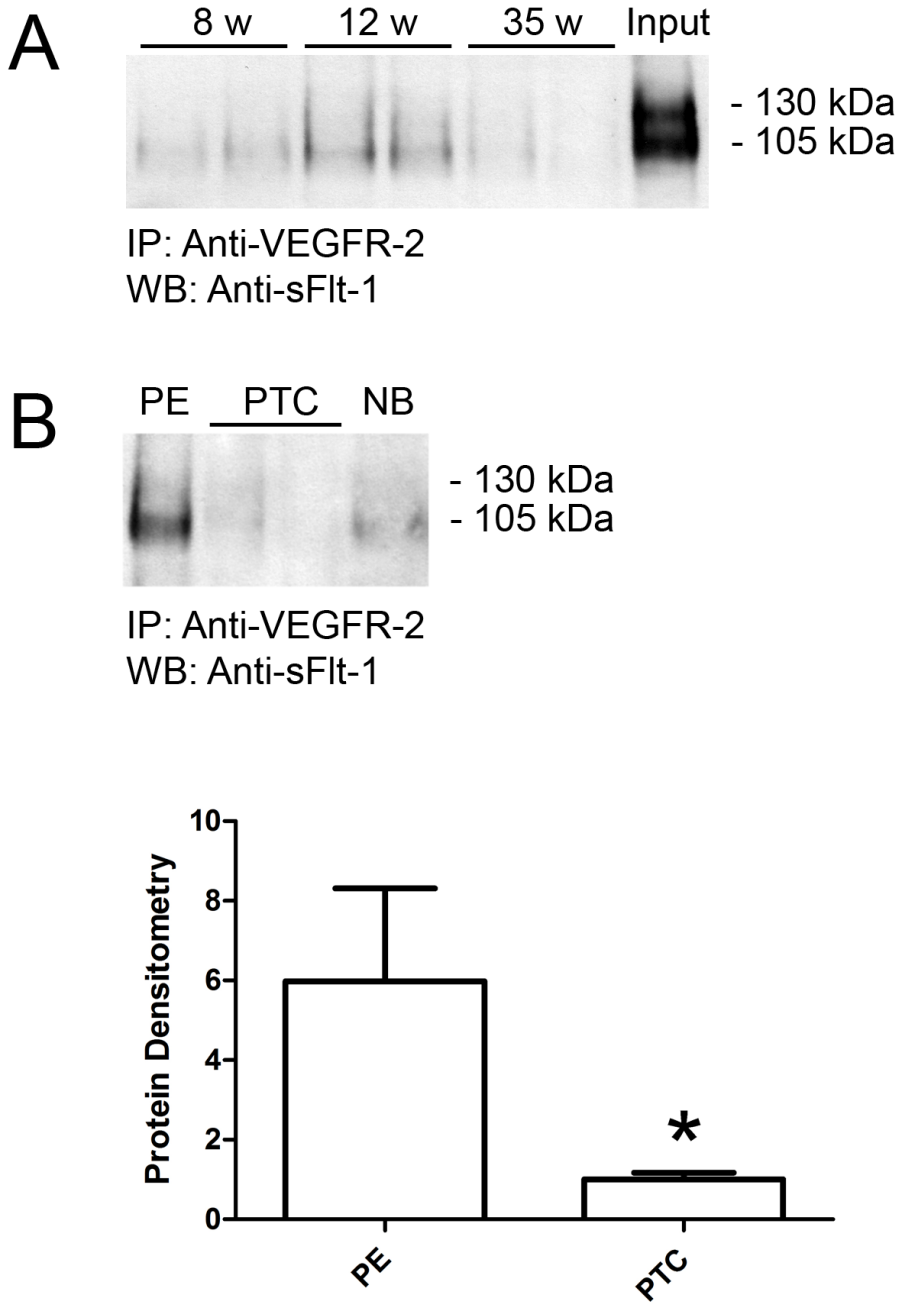
Increased association of VEGFR-2 and sFlt-1 in preeclamptic placentae. **A:** Representative sFlt-1 immunoblot of developmental placental samples (two different tissue samples at each gestational age of 8, 12 and 35 weeks) immunoprecipitated with a monoclonal VEGFR-2 antibody. A control lane of protein lysates prepared from placental tissue (35 weeks of gestation) is indicated (Input). **B:** Representative sFlt-1 immunoblot of PE and PTC samples immunoprecipitated with a monoclonal VEGFR-2 antibody. A negative control lane of PE protein lysate immunoprecipitated in the absence of VEGFR-2 antibody is indicated (NB). Densitometry analysis comparing sFlt-1 immunoreactivity between PE (n = 3) and PTC (n = 6) samples immunoprecipitated with VEGFR-2 antibody. (IP, immunoprecipitation antibody; WB, western blot antibody; PE, preeclampsia; PTC, age-matched preterm control; *P<0.05). All values are represented as the means±SEM.

With the degree of co-localization observed between VEGFR-2 and sFlt-1 in the vasculature of placentae from PE patients, we speculated that VEGFR-2 and sFlt-1 may exhibit increased levels of association or heterodimerization in PE. We first examined the extent of VEGFR-2 and sFlt-1 association at various stages of placental development (8, 12 and 35 weeks of gestation). Protein lysates were prepared from each gestational age and immunoprecipitated for VEGFR-2 and subjected to western analysis for sFlt-1. While some levels of association were observed for 8 and 35 weeks, the strongest immunoreactive sFlt-1 signals were observed at 12 weeks of gestation (Fig. 4A). While both expected forms of sFlt-1 were observed at 130 and 105 kDa in the lysate input, the 105 kDa species was the predominant form associated with VEGFR-2.

We next assessed the association of VEGFR-2 and sFlt-1 in PE and PTC placentae (Fig. 4B). PTC lysates immunoprecipitated for VEGFR-2 yielded similar results to the 35 week samples with minimal levels of co-immunoprecipitated sFlt-1. However, PE lysates immunoprecipitated for VEGFR-2 revealed a strong immunoreactive sFlt-1 band of 105 kDa, demonstrating a greater association of VEGFR-2 and sFlt-1 in PE placentae. Densitometry analysis confirmed this association was significantly higher in PE over PTC placentae 5.98 fold (5.98±2.34 versus 1.00±0.17, *P*<0.05).

### sFlt-1 directly attenuates VEGFR-2 expression

**Figure 5:**
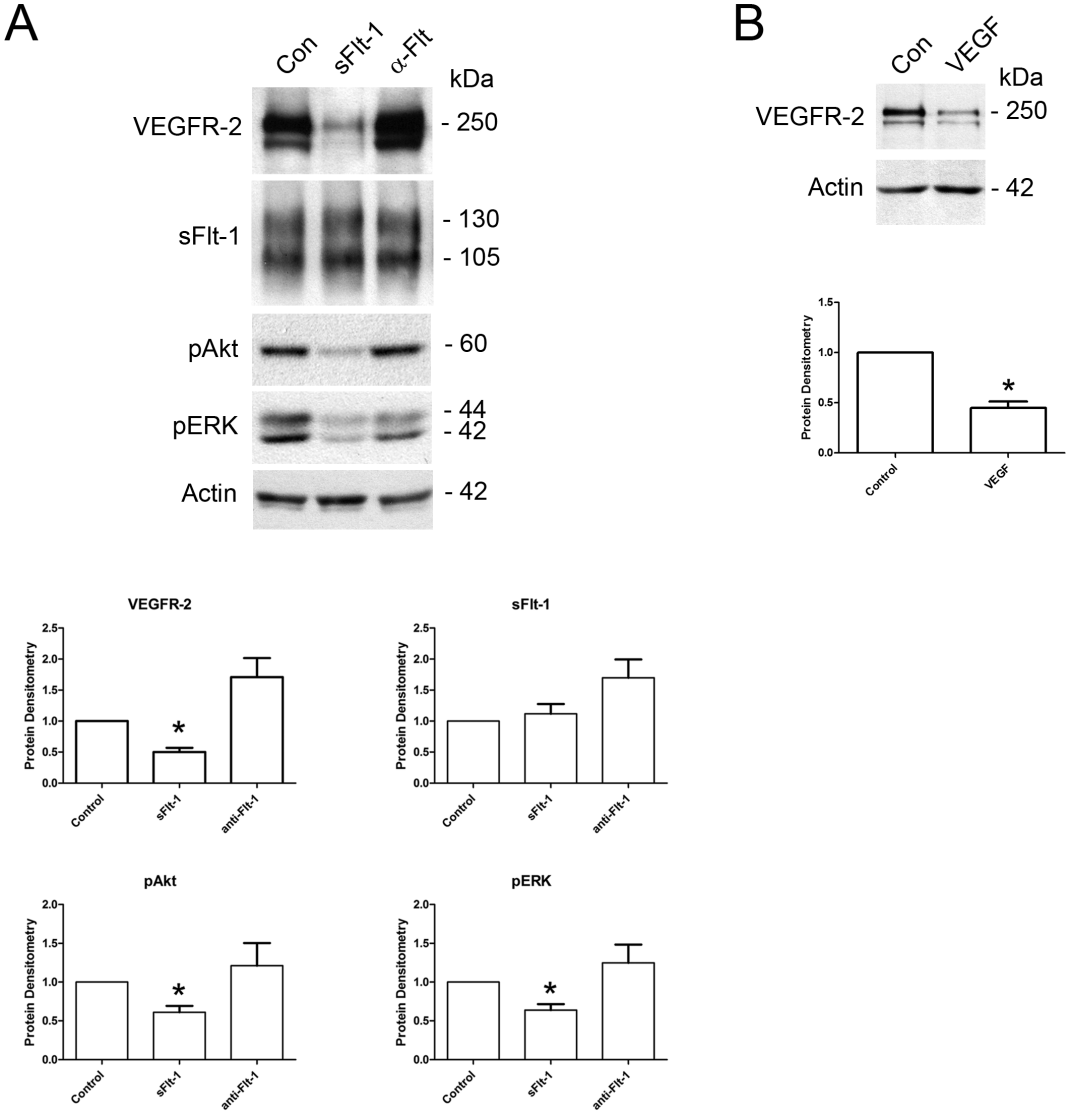
sFlt-1 directly attenuates VEGFR-2 expression and downstream signalling in placental villous explants. **A:** Representative VEGFR-2, sFlt-1, pAkt and pERK immunoblots of first trimester placental villous explants treated with sFlt-1 or a blocking antibody to sFlt-1. Densitometry analyses comparing normalized VEGFR-2, sFlt-1, pAkt and pERK in treated samples (Con, control; sFlt-1, sFlt-1 protein; α-Flt, Flt-1 neutralizing antibody; n = 3, *P<0.05). All values are represented as the means±SEM. **B:** Representative VEGFR-2 immunoblot of first trimester placental villous explants treated with VEGF. Densitometry analyses comparing normalized VEGFR-2 between control and VEGF-treated samples (Con, control; VEGF, VEGF-treated; n = 3, *P<0.05). All values are represented as the means±SEM.

With reciprocal levels of expression and increased co-localization and association of VEGFR-2 and sFlt-1 in PE placentae, we hypothesized that sFlt-1 might directly abate VEGFR-2 levels. First trimester placental villous explants were cultured in 20% O_2_ and treated with 500 ng/mL sFlt-1 for 17 hours. Protein lysates were prepared and subjected to western analyses for VEGFR-2, sFlt-1, phosphorylated-ERK (pERK) and phosphorylated-Akt (pAkt) levels (Fig. 5A). Compared to untreated control explants, those treated with sFlt-1 exhibited significantly decreased levels of VEGFR-2 (2.03 fold) and decreased downstream signaling proteins pERK (1.60 fold) and pAkt (1.64 fold), confirming a direct negative regulation of VEGFR-2 expression and signalling by sFlt-1. Endogenous sFlt-1 levels were not significantly altered. In addition, we attempted to counteract any effects of endogenous sFlt-1 secreted into the media by the explants by overnight administration of a monoclonal antibody to Flt-1 (600 ng/mL). While western analyses revealed consistent trends of increased VEGFR-2, sFlt-1, pERK and pAkt, these results were not statistically significant. To confirm that decreased levels of VEGFR-2 did not arise from sFlt-1 sequestration of VEGF in the media, explants were also treated with 20 ng/mL VEGF for 17 hours (Fig. 5B). VEGF-treated explants exhibited significantly decreased VEGFR-2 levels, which suggests that VEGF sequestration by sFlt-1 would stabilize if not increase VEGFR-2 levels.

## Discussion

VEGFR-2 is a receptor tyrosine kinase that is expressed in endothelial cells and in the trophoblast layer of the human placenta; however, its regulation of expression in the human placenta has not yet been reported. Placental hypoxia and increased levels of sFlt-1 are implicated in placental disorders such as preeclampsia and therefore we investigated whether they affect placental VEGFR-2 expression. We found that VEGFR-2 expression is reduced in placentae from preeclamptic patients and is inversely related to the levels of sFlt-1 in the same samples. Hypoxia induces a profound decrease in VEGFR-2 expression in the human placenta both in-vivo and in-vitro. sFlt-1 directly interacts with VEGFR-2 in-vivo and reduces VEGFR-2 expression and its downstream signalling in placental villous explants. Altogether, our findings suggest a paramount role for sFlt-1 in VEGFR-2 activity, through heterodimerization with VEGFR-2 and subsequent inhibition of VEGFR-2 expression and signaling in placental trophoblasts and endothelial cells.

The expression of VEGFR2 in the human placenta from both normal and preeclamptic pregnancies has been previously studied, though a consensus has yet to be reached in terms of distribution and relative levels. Helske et al. found that VEGFR2 was expressed almost exclusively in the endothelial cells of fetal placental vasculature and there was no difference in staining intensity between normal and preeclamptic samples [20]. However, Marini et al. found lower protein levels of VEGFR-2 in placental vasculature in both preeclampsia and HELLP compared with controls [21]. Other reports describe a clear trophoblast localization of VEGFR-2, including cytotrophoblasts, the proximal column, endovascular cytotrophoblasts [10] and in all populations of extravillous trophoblasts in placental beds during normal pregnancy [22]. Overall, our findings support the work by Zhou et al. [10] as we found VEGFR-2 expression in cytotrophoblasts, proximal column, and placental vasculature. Additionally, we observed that VEGFR-2 protein and mRNA are reduced in placentae from preeclamptic patients and when comparing its expression to sFlt-1 level in the same sample we found a surprising inverse correlation. This in turn suggests that sFlt-1 may interfere with VEGFR-2 expression or an underlying process that induces both an increase in sFlt-1 and decrease in VEGFR-2 expression.

VEGFR-2 expression in endothelial cells has been previously shown to decrease under hypoxic conditions though the evidence is somewhat conflicting. VEGFR-2 expression was observed to decrease during hypoxia in human coronary endothelial cells [11] and in HUVECs [23]. However, others have reported that HUVECs exposed to hypoxic conditions resulted in unchanged or only mildly decreased expression [24] or even up-regulation of VEGFR-2 [25]. Here we present the first evidence that VEGFR-2 expression in the human placenta is substantially decreased in both the protein and RNA levels under hypoxic conditions and thus support the more recent studies [11], [23]. The in-vitro model of villous explants that was used here is different from previous work that focused on endothelial cells since villous explants include two cell types that express VEGFR-2: trophoblasts and endothelial cells. We have shown by immunolocalization that VEGFR-2 expression in villous explants is present mainly in trophoblasts and is decreased during hypoxia in a similar fashion to what was found in endothelial cells, suggesting that most of the changes we observed can be attributed to decreased expression in the trophoblast layer. Our in-vitro observation is also supported by in-vivo data showing reduced placental VEGFR-2 expression during the very early first trimester which is characterised by low oxygen levels.

Homodimerization of VEGFR-2 is crucial for its activation through phosphorylation of the intracellular domains while heterodimerization of VEGFR-2 and VEGFR-1 has been shown to promote intracellular downstream signaling of the VEGFR-2 receptor and facilitate migration of endothelial cells [6], [26]. Moreover, VEGFR-1 and VEGFR-2 heterodimers were found to have unique signaling properties which may be related to specific Ca^2+^ release [26]. Recently, Cudmore et al. reported that activation of heterodimers between VEGFR-1 and VEGFR-2 mediates VEGFR-2 phosphorylation, endothelial cell migration, tube formation and secretion of nitric oxide but not endothelial cell proliferation or tissue factor expression which are facilitated by VEGFR-2 homodimers [27], supporting the importance of VEGFR-1 and VEGFR-2 heterodimerization in endothelial cell homeostasis. In addition, heterodimerization between sFlt-1 and VEGFR-2 in-vitro was reported by Kendall et al., who found that sFlt-1 can form a negative complex with VEGFR-2, thus inhibiting its function [7]. Our findings support that sFlt-1 associate with VEGFR-2 in-vivo and form a negative heterodimer as was suggested by Kendall et al. This inhibitory function of sFlt-1 may be a consequence of inhibiting both the homo and heterodimerization of VEGFR-2 by direct binding to VEGFR-2 and thus blocking it from forming dimers, leading to decreased intracellular signaling of the receptor. Alternatively, sFlt-1 interaction/heterodimerization with VEGFR-2 may facilitate VEGFR-2 degradation or internalization which was previously shown to occur after VEGF exposure [28]. Zhang et al. showed that VEGF treatment which promote dimerization of VEGFR-2 induces VEGFR-2 trafficking and ubiquitination with no change at the transcriptional level of VEGFR-2. This process is also associated with endocytosis, though VEGFR-2 downregulation was primarily due to ubiquitination [28].

sFlt-1 levels are markedly elevated in-vivo only in preeclampsia, which is a unique human disorder and previous in-vivo studies have not shown this phenomenon in humans until now. Here, using human placental tissue, we found that sFlt-1 directly interacts with VEGFR-2 in-vivo, and this phenomenon is significantly more prominent in placental tissue from preeclamptic patients, which is known to express higher levels of sFlt-1. We speculate that heterodimerization of sFlt-1 and VEGFR-2 may contribute to the reduced levels of VEGFR-2 in placenta from preeclamptic patients, and may thus explain the inverse correlation between higher levels of sFlt-1 and lower levels of VEGFR-2 in placentae from preeclamptic patients.

Increased sFlt-1 levels are associated with lower levels of the free ligands VEGF and PlGF [1], while increased VEGF level has been shown here and also before to decrease the level of VEGFR-2 [28]. Therefore, while sFlt-1 could hypothetically increase VEGFR-2 level by decreasing the levels of the two ligands (VEGF and PlGF), we have found that sFlt-1 instead reduces VEGFR-2 levels, suggesting that VEGF levels do not play a role in this process.

There are several known isoforms of sFlt-1 in human placenta including the well known sFlt-13 that includes a short unique sequence from intron 13 and sFlt-14 that is longer and includes a fraction of intron 14 [3], [4]. We found that sFlt-13 with molecular weight of about 100-105 Kd is the main isoform that forms heterodimers with VEGFR-2 while the level of sFlt-14 heterodimerization was minimal. The reason for the preferential sFlt-13 heterodimerization with VEGFR-2 is not completely clear though we can speculate that the addition of exon/intron 14 interferes with the heterodimerization process.

In summary, our findings suggest a direct inhibitory effect of sFlt-1 on VEGFR-2 expression and signalling in human placentae. The distribution and relative levels of sFlt-1 and VEGFR-2 in placentae reveal an inverse correlation between the related receptors, with decreased levels of VEGFR-2 associated with increased levels of sFlt-1, hypoxia and preeclampsia. Furthermore, increased levels of co-immunoprecipitation between sFlt-1 and VEGFR-2 in preeclamptic placentae suggests a direct inhibitory effect of sFlt-1 on VEGFR-2, which was confirmed by decreased levels of VEGFR-2 and downstream signalling proteins in villous explants treated with exogenous sFlt-1. In addition to current hypotheses that speculate on the inhibitory effects of sFlt-1 on angiogenesis and endothelial function by sequestration of VEGF and PlGF, these studies propose another, and more direct, route of action – an inhibitory heterodimerization complex between sFlt-1 and VEGFR-2.
